# Karyotypes of parasitic wasps of the family Eulophidae (Hymenoptera) attacking leaf-mining Lepidoptera (Gracillariidae, Gelechiidae)

**DOI:** 10.3897/CompCytogen.v8i1.6537

**Published:** 2014-02-25

**Authors:** Vladimir E. Gokhman, Zoya A. Yefremova, Ekaterina N. Yegorenkova

**Affiliations:** 1 Botanical Garden, Moscow State University, Moscow 119234, Russia; 2 Department of Zoology, The George S. Wise Faculty of Life Sciences, Tel Aviv University, Tel Aviv 69978, Israel; 3 Department of Geography, Ulyanovsk State Pedagogical University, Ulyanovsk 432700, Russia

**Keywords:** Hymenoptera, Chalcidoidea, Eulophidae, Lepidoptera, Gracillariidae, Gelechiidae, chromosomes, karyotypes, B chromosomes

## Abstract

Karyotypes of eleven parasitoid species of the family Eulophidae were examined, namely, *Chrysocharis laomedon* (Walker, 1839) (2n = 10), *Chrysocharis* sp. aff. *laomedon* (n = 5, 2n = 10), *Chrysocharis* sp. aff. *albipes* (Ashmead, 1904) (2n = 12), *Mischotetrastichus petiolatus* (Erdös, 1961) (n = 6, 2n = 12), *Minotetrastichus frontalis* (Nees, 1834) (n = 5, 2n = 10), *Cirrospilus pictus* (Nees, 1834) (2n = 12), *Hyssopus geniculatus* (Hartig, 1838) (2n = 16), *Sympiesis gordius* (Walker, 1839) (2n = 12), *S. sericeicornis* (Nees, 1834) (2n = 12), *Pnigalio agraules* (Walker, 1839) (2n = 12 + 0–2B) and *Pnigalio gyamiensis* Myartseva & Kurashev, 1990 (2n = 12 + 0–6B) reared from *Phyllonorycter acerifoliella* (Zeller, 1839), *Ph. apparella* (Herrich-Schäffer, 1855), *Ph. issikii* (Kumata, 1963) (Gracillariidae) and *Chrysoesthia sexguttella* (Thunberg, 1794) (Gelechiidae). Chromosome sets of all species except *P. agraules* and *P. gyamiensis* were studied for the first time. B chromosomes were detected in the two latter species; in *P. gyamiensis*, the maximum number of B chromosomes represents the highest value known for parasitic wasps to date.

## Introduction

The Eulophidae are one of the largest and most diverse families of the hymenopteran superfamily Chalcidoidea. This group currently contains about 300 genera and 4500 described species (Noyes 2013). Chromosomal study of these parasitoids is becoming a rapidly developing research field due to its implications for taxonomy and evolutionary history of this morphologically challenging group, with about 60 species of the family Eulophidae (i.e. more than 1% of described species) karyotyped up to now. Specifically, two reviews of Eulophidae karyology were published in the 2000s (Gokhman 2002, 2004), and a number of other papers on this subject appeared since that time (e.g. Gebiola et al. 2012a, Gokhman and Gumovsky 2013, and references therein) including a monograph on karyology of hymenopteran parasitoids (Gokhman 2009). Furthermore, certain members of the family have become objects of an advanced cytogenetic study (Bolsheva et al. 2012). We have recently examined chromosome sets of a number of Eulophidae species associated with leaf-mining Lepidoptera of the families Gracillariidae and Gelechiidae. This group of the family Eulophidae has been chosen for the present study because its members are well known as the most abundant parasitoids of leaf-mining Lepidoptera (see e.g. Yefremova and Mishchenko 2008, Yefremova et al. 2009, 2011). At the same time, many species of these parasitic wasps belong to the subfamily Eulophinae that is, in turn, the most karyotypically diverse group of the Eulophidae (Gokhman 2002, 2009).

## Material and methods

The material used in this study was collected by V.E. Gokhman and E.N. Yegorenkova in the Moscow (Ozhigovo, 60 km SW Moscow; 55°27'N, 36°52'E) and Ulyanovsk Provinces (Ulyanovsk; 54°16'N, 48°20'E) of Russia in 2012–2013 respectively (Table 1). All parasitoids were reared from *Phyllonorycter acerifoliella* (Zeller, 1839) on *Acer platanoides* Linnaeus, *Phyllonorycter apparella* (Herrich-Schäffer, 1855) on *Populus tremula* Linnaeus, *Phyllonorycter issikii* (Kumata, 1963) (Gracillariidae) on *Tilia cordata* Miller and *Chrysoesthia sexguttella* (Thunberg, 1794) (Gelechiidae) on *Chenopodium album* Linnaeus. Preparations of mitotic chromosomes (as well as meiotic chromosomes where available) of all species except *Minotetrastichus frontalis* were obtained from ovaries of adult females according to the protocol developed by Gokhman (2009) with minor modifications. Specifically, the extracted ovaries were incubated in 0.5% hypotonic colchicine-added sodium citrate solution for 30 min (the original technique implies incubation in 1% hypotonic solution for 20 min). Alternatively, chromosomes of gregarious *Minotetrastichus frontalis* were studied on preparations of cerebral ganglia of prepupae according to the slightly modified technique developed by Imai et al. (1988). Again, above-specified incubation parameters were used; a few individuals from some broods were reared to the adult stage and then identified. Numbers of examined specimens as well as mitotic and meiotic divisions for each species are given in Table 1. Cell divisions were studied and photographed using an optic microscope Zeiss Axioskop 40 FL fitted with a digital camera AxioCam MRc. To obtain karyograms, the resulting images were processed with image analysis programs Zeiss AxioVision version 3.1 and Adobe Photoshop version 8.0. Mitotic chromosomes were subdivided into four groups: metacentrics (M), submetacentrics (SM), subtelocentrics (ST) and acrocentrics (A) following guidelines provided by Levan et al. (1964) and Insua et al. (2006). For species with already known karyotypes, morphometric analysis of chromosomal morphology was performed. Selected metaphase plates with the clearly visible centromeric position of every chromosome were measured using Adobe Photoshop; relative lengths and centromeric indices of all chromosomes were calculated and given in Table 2. Meiotic chromosomes were classified according to Darlington (1965). Parasitoids were identified by Z.A. Yefremova (Eulophinae and Entedoninae) and E.N. Yegorenkova (Tetrastichinae) using keys provided by Trjapitzin (1978) and Storozheva et al. (1995), Hansson (1985) as well as by Graham (1987) respectively; however, most individuals belonging to the taxonomically complicated genus *Chrysocharis* Förster, 1856 could not be reliably assigned to any named species. Voucher specimens are deposited in the Zoological Museum, Moscow State University, Moscow, Russia.

**Table 1.** The main results of karyotypic study of the Eulophidae (Hymenoptera) attacking leaf-mining Lepidoptera.

Species	Origin		Number		(n), 2n	Chromosomal formula: (n), 2n
	Locality	Host	(Males), females	(Meiotic), mitotic divisions		
*Chrysocharis laomedon*	Ozhigovo	*Phyllonorycter issikii*	1	11	10	10M
*Chrysocharis* sp. aff. *laomedon*	Ozhigovo, Ulyanovsk	*Phyllonorycter acerifoliella*, *Phyllonorycter issikii*	7	(3), 13	(5), 10	10M
*Chrysocharis* sp. aff. *albipes*	Ozhigovo	*Phyllonorycter apparella*	1	10	12	10M + 2ST
*Mischotetrastichus petiolatus*	Ditto	*Phyllonorycter issikii*	2	(4), 2	(6), 12	10M + 2A
*Minotetrastichus frontalis*	Ulyanovsk	Ditto	(1), 7	15	(5), 10	(5M), 10M
*Cirrospilus pictus*	Ozhigovo	*Phyllonorycter apparella*	2	6	12	6M + 2SM + 4ST
*Hyssopus geniculatus*	Ditto	*Phyllonorycter issikii*	3	14	16	6M + 2SM + 4ST + 4A
*Sympiesis gordius*	Ditto	Ditto	2	2	12	12M
*Sympiesis sericeicornis*	Ditto	*Phyllonorycter apparella*	2	10	12	10M + 2A
*Pnigalio agraules*	Ditto	*Phyllonorycter apparella*, *Phyllonorycter issikii*	3	13	2n = 12 + 0–2B	10M + 2M/SM
*Pnigalio gyamiensis*	Ulyanovsk	*Chrysoesthia sexguttella*	1	17	2n = 12 + 0–6B	6M + 4M/SM + 2ST

**Table 2.** Relative lengths (RL) and centromeric indices (CI) of *Pnigalio agraules* and *Pnigalio gyamiensis* chromosomes (mean ± SD; B chromosomes not included). For each species, numbers of analyzed metaphase plates are given in brackets.

Chromosome no.	*Pnigalio agraules* (6)		*Pnigalio gyamiensis* (8)	
	RL	CI	RL	CI
1	21.09 ± 1.09	44.74 ± 3.50	20.44 ± 0.75	45.43 ± 3.37
2	18.89 ± 0.84	43.32 ± 3.56	19.06 ± 0.85	40.87 ± 5.13
3	17.31 ± 0.70	41.82 ± 3.69	18.04 ± 0.54	41.40 ± 4.59
4	16.06 ± 0.61	44.40 ± 4.63	16.69 ± 0.76	44.82 ± 3.79
5	14.37 ± 0.83	45.18 ± 3.92	14.18 ± 0.93	45.50 ± 4.61
6	12.28 ± 1.42	38.39 ± 5.36	11.59 ± 1.24	18.82 ± 5.34

## Results

The principal results of the present study are listed in Table 1; some additional details are given below.

### Subfamily Entedoninae

**Figure 1.**
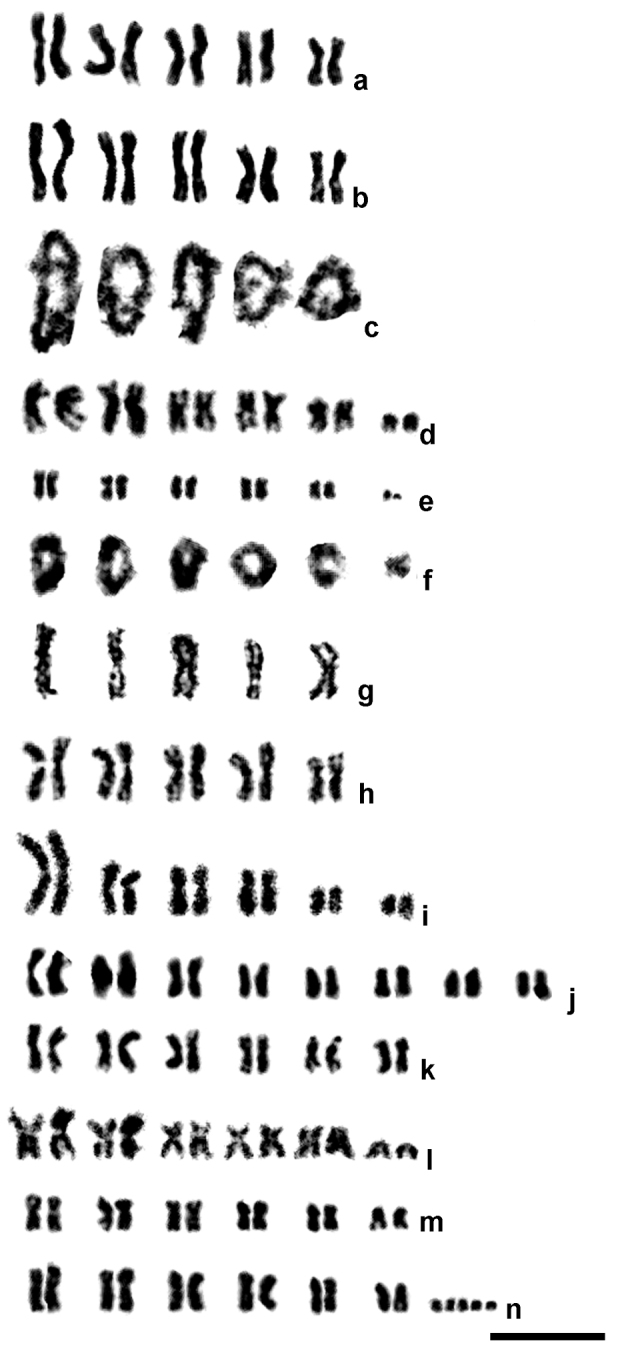
Mitotic (**a–b, d–e, g–n**) and meiotic (diplotene; **c, f**) karyograms of male (**g**) and female (**a–f, h–n**) parasitic wasps of the family Eulophidae. **a** *Chrysocharis laomedon* **b–c** *Chrysocharis* sp. aff. *laomedon* **d** *Chrysocharis* sp. aff. *albipes* **e–f** *Mischotetrastichus petiolatus* **g–h** *Minotetrastichus frontalis* **i** *Cirrospilus pictus* **j** *Hyssopus geniculatus* **k** *Sympiesis gordius* **l** *Sympiesis sericeicornis* **m** *Pnigalio agraules* **n** *Pnigalio gyamiensis*, karyotype with five B chromosomes. Bar = 10 µm (6.7 µm for **c**).

*Chrysocharis laomedon* (Walker, 1839) (Fig. 1a). All chromosomes are obviously metacentric; chromosomes of the first, second and third pair, and those of the fourth and fifth pair, form three size groups.

*Chrysocharis* sp. aff. *laomedon* (Fig. 1b–c). Karyotype structure of the mitotic chromosome set as in *Chrysocharis laomedon* (Fig. 1b). The meiotic karyotype contains five bivalents; each of them apparently bears two chiasmata in diplotene (Fig. 1c).

*Chrysocharis* sp. aff. *albipes* (Ashmead, 1904) (Fig. 1d). As in the two previous species, chromosomes of the five largest pairs are metacentric, but a pair of small subtelocentrics is present in the karyotype as well. The metacentrics also form three size groups; however, apart from previous species, these groups include chromosomes of the first and second, third and fourth, and fifth pair respectively.

### Subfamily Tetrastichinae

*Mischotetrastichus petiolatus* (Erdös, 1961) (Fig. 1e–f). The karyotype contains five pairs of metacentric chromosomes; the first and the last pair are visibly longer/shorter respectively than the remaining ones. In addition, a pair of small acrocentrics is present in the chromosome set (Fig. 1e). Six bivalents are found in the meiotic karyotype of this species; in diplotene, almost all of them bear two chiasmata except for the last one with a single chiasma (Fig. 1f).

*Minotetrastichus frontalis* (Nees, 1834) (Fig. 1g–h). Both haploid (Fig. 1g) and diploid karyotypes (Fig. 1h) were studied. All chromosomes are obviously metacentric; those of the fifth pair are substantially smaller than chromosomes of the preceding ones.

### Subfamily Eulophinae

*Cirrospilus pictus* (Nees, 1834) (Fig. 1i). Metacentrics of the first pair very large, at least more than 1.5 times longer than the remaining chromosomes. Metacentrics of the second and third pair as well as submetacentrics of the fourth pair more or less gradually decrease in length. Subtelocentric chromosomes of the fifth and sixth pair substantially differ in size and visibly smaller than the preceding ones.

*Hyssopus geniculatus* (Hartig, 1838) (Fig. 1j). The diploid chromosome number in this species is substantially higher than in many other members of the family. First three chromosome pairs obviously differ in size and are somewhat longer than the remaining ones. The karyotype contains metacentric (the first, third and eighth pair), submetacentric (the second pair), subtelocentric (the fourth and sixth pair) and acrocentric chromosomes (the fifth and seventh pair).

*Sympiesis gordius* (Walker, 1839) (Fig. 1k). All chromosomes are metacentric; metacentrics of the first pair substantially differ in size from the remaining ones. Chromosomes of the fifth pair bear distinct secondary constrictions.

*Sympiesis sericeicornis* (Nees, 1834) (Fig. 1l). The karyotype contains five pairs of large metacentric chromosomes and a small pair of acrocentrics; chromosomes of the first pair are visibly longer than the other metacentrics.

**Figure 2.**
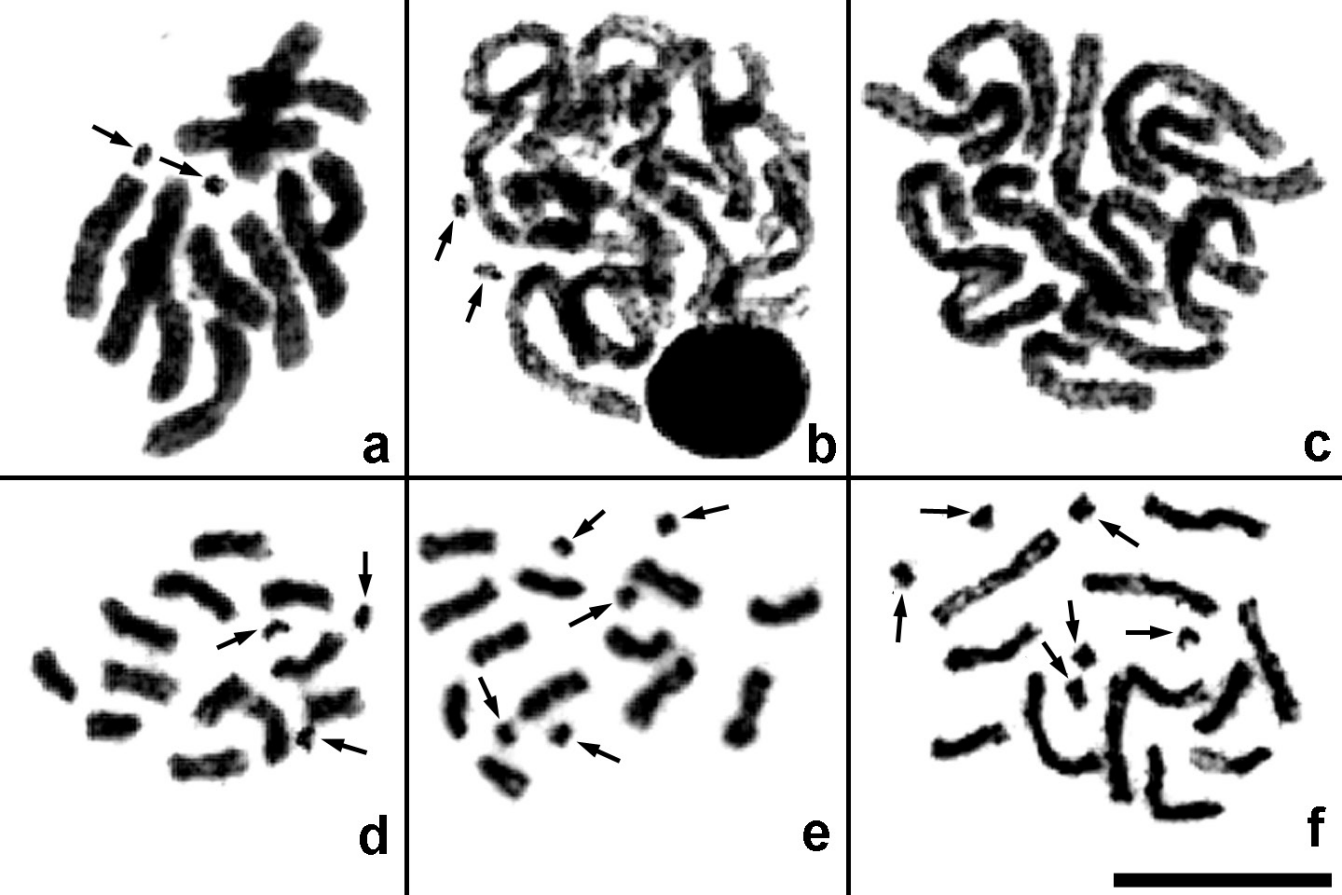
Mitotic divisions in *Pnigalio agraules* (**a–c**) and *Pnigalio gyamiensis* (**d–f**). **a** Fragment of a metaphase plate with two B chromosomes **b** Same individual, late prophase with two B chromosomes **c** Same individual, prometaphase without B chromosomes **d–f** Metaphase plates with three, five and six B chromosomes respectively. Arrows indicate B chromosomes. Bar = 10 µm.

*Pnigalio agraules* (Walker, 1839) (Figs 1m, 2a–c). The first and the last chromosome pair are obviously longer/shorter respectively than the remaining ones that form a continuous gradation in length (Table 2). Most chromosomes are metacentric except for the sixth pair that can be either metacentric or submetacentric (Fig. 1m). In addition, a single specimen carrying B chromosomes was found. Although we were unable to obtain full metaphase plates in the former individual, fragments of these plates (Fig. 2a) as well as certain mitotic divisions in late prophase or early metaphase (Fig. 2b) clearly demonstrate presence of the two B chromosomes. Nevertheless, other cell divisions from the same individual show no trace of the chromosomes of that kind (Fig. 2c).

*Pnigalio gyamiensis* Myartseva & Kurashev, 1990 (Figs 1n, 2d–f). The overall karyotype structure as in the preceding species (Table 2), but the second and third chromosome pair can be either metacentric or submetacentric, and the last chromosome pair is subtelocentric (Fig. 1n). In addition, most metaphase plates carry a few very small apparently subtelocentric or acrocentric B chromosomes (usually two to five, but sometimes one or six; Figs 1n, 2d–f).

## Discussion

The family Eulophidae is the most karyotypically studied group of parasitoids of its taxonomic rank in terms of the relative number of studied species. The results presented here provide new information on chromosome number and morphology in certain groups of the family. All species listed in the present paper (except for *Pnigalio agraules* and *Pnigalio gyamiensis*; see below) as well as the genera *Mischotetrastichus* Graham, 1987, *Minotetrastichus* Kostjukov, 1977 and *Chrysocharis* Förster, 1856 were studied for the first time. In addition, new karyotypic information was obtained for the genus *Cirrospilus* Westwood, 1832. Within this genus, only the chromosome number was studied earlier for *Cirrospilus diallus* Walker, 1838 (n = 6; Gokhman and Quicke 1995). Specifically, the haploid chromosome number in the species studied varies from n = 5 to n = 8. Interestingly, this variation range is also observed in the family Eulophidae in general (Gokhman 2002). The new data confirm our previous conclusion that the haploid set of five long bi-armed chromosomes and a short acro- or subtelocentric represents the ancestral feature of the Eulophidae (Gokhman 2002, 2004), possibly a synapomorphy for the family (Gokhman 2009). Since many Torymidae (together with a few Ormyridae) as well as certain Agaonidae also have similar karyotypes, this feature is likely to have been independently acquired by various groups of the superfamily Chalcidoidea (Gokhman 2013).

Nevertheless, a few deviations from the above mentioned pattern have been recorded up to now, including “*Elachertus* sp.” with n = 8 (Gokhman 2002, 2009) and *Cirrospilus pictus* with n = 6 in the present study. As for the former, this aberrant chromosome number together with a characteristic karyotype structure (see Fig. B.207 in Gokhman 2009) was detected in a single specimen identified by V.V. Kostjukov. Since the same n value was found during the present study in *Hyssopus geniculatus*, we have re-examined the former specimen that is also deposited in the Zoological Museum of Moscow State University. In fact, this time it was identified by Z.A. Yefremova as a member of the genus *Hyssopus* Girault, 1916, namely *Hyssopus nigritulus* (Zetterstedt, 1838). *Hyssopus* was actually placed within *Elachertus* Spinola, 1811 as its subgenus or a separate species group for a certain period (e.g. Bouček 1965). The aberrant karyotype structure found in both studied *Hyssopus* species can therefore be considered as a autapomorphy of this genus. On the other hand, the chromosome set of *Cirrospilus pictus* (n = 6) represents a particular karyotype structure that was previously unknown in the Eulophidae. In addition, chromosomes of the last pair in *Pnigalio agraules* and *Pnigalio gyamensis* with the same haploid number are substantially longer than those characteristic of the common karyotype structure in the family (see also Gebiola et al. 2012a).

Chromosomal rearrangements involved in the karyotype evolution of the Eulophidae possibly include chromosomal fusions in a few species (e.g. *Minotetrastichus frontalis* and *Chrysocharis laomedon*). In these cases, the smallest chromosome fused to one of the larger elements to form a karyotype with n = 5. On the other hand, an increase in chromosome number in certain groups (e.g. in the genus *Hyssopus*) could take place by aneuploidy and the subsequent restoration of even chromosome numbers (Gokhman 2009). An alternative explanation, i.e. chromosomal fissions followed by inversions (see e.g. Imai et al. 1988), seems less likely, mainly due to the lack of smaller chromosomes that could arise from these rearrangements in *Hyssopus* and a few other Eulophidae.

Karyotype structure found in *Mischotetrastichus* and *Minotetrastichus* showed certain resemblance to that of *Tetrastichus* Haliday, 1844 s.str. Specifically, metacentrics of the last pair are substantially shorter than the preceding ones (Gokhman 2004).

The meiotic figures obtained in *Chrysocharis* sp. aff. *laomedon* and *Mischotetrastichus petiolatus* generally correspond to mitotic karyotypes of these parasitoids. Specifically, bivalents apparently formed by metacentric chromosomes are relatively large and gradually decrease in size. These ring-like bivalents bear two terminal/subterminal chiasmata in diplotene. Alternatively, the only acrocentric pair found in *Mischotetrastichus petiolatus* forms a small open bivalent with a single chiasma.

We have also detected B chromosomes in *Pnigalio agraules* and *Pnigalio gyamiensis*. Interestingly, chromosome sets of both members of this genus have been recently examined by Gebiola et al. (2012a), with the latter species listed there as *Pnigalio soemius* (Walker, 1839) (i.e. *P. soemius*_CS; see Gebiola et al. 2012b). Overall karyotype structure of the two *Pnigalio* Schrank, 1802 species studied by Gebiola et al. (2012a) generally coincides with our results except for the last chromosome pair of *Pnigalio gyamiensis* which appeared to be subtelocentric according to the present study. However, Gebiola et al. (2012a) could define this pair as acrocentric arbitrarily, since no quantitative data on its centromeric position are given in the cited paper. Moreover, no B chromosomes were previously detected in both members of the genus *Pnigalio*. Up to now, chromosomes of that kind were found only in a few species of chalcid wasps belonging to the families Pteromalidae, Trichogrammatidae and possibly also Aphelinidae (see Gokhman 2009 for review), and Eulophidae (Gebiola et al. 2012a). In all those cases, only one chromosome per karyotype was detected. In addition, chromosomes of a particular pair found in *Aphidius ervi* Haliday, 1834 (Braconidae) with 2n = 12 (Gokhman and Westendorff 2003) can also be considered, with certain reservations, as B chromosomes. Gebiola et al. (2012a) found B chromosomes in the genus *Pnigalio*, i.e. in *Pnigalio mediterraneus* Ferrière & Delucchi, 1957. Again, karyotypes of a few individuals of this species carried the only B chromosome. We found two B chromosomes in the karyotype of a certain female of *Pnigalio agraules*. However, identity of the latter species poses a separate problem. Indeed, *Pnigalio mediterraneus* and *Pnigalio agraules* cannot be reliably separated on the basis of adult external morphology alone (Gebiola et al. 2009). Nevertheless, karyotype of the former species contains strictly metacentric chromosomes, whereas certain chromosomes of *Pnigalio agraules* can be submetacentric (Gebiola et al. 2012a), and this is characteristic of our specimens as well. On the other hand, borders between closely related taxa (perhaps even between different genera) are probably not impermeable for B chromosomes, as it was suggested for an analogous chromosome found in the pteromalid *Nasonia vitripennis* (Walker, 1836) (McAllister and Werren 1997). We also found one to six B chromosomes in *Pnigalio gyamiensis*. The latter value therefore represents the highest number of B chromosomes per diploid karyotype known for parasitic wasps to date. As far as other members of the order Hymenoptera are concerned (see Gokhman 2009 for a brief review), up to 12 chromosomes per haploid set were found in the ant, *Leptothorax spinosior* Forel, 1901 (Imai 1974), which is one of the highest records in the animal world (Camacho et al. 2000). Although B chromosomes often carry sex-ratio distorters in parasitoid Hymenoptera, as opposed to the aculeate members of the order (Gokhman 2009), this is probably not the case in *Pnigalio gyamiensis*, thus providing a possible explanation for accumulation of these chromosomes in the latter species.

The present research also revealed differences between karyotypes of closely related taxa (e.g. within the genus *Chrysocharis*), thus confirming that chromosomal studies can be used for identifying cryptic species in the Eulophidae, as in many other parasitoid families (Gokhman 2009, Gebiola et al. 2012a). Furthermore, data provided in the present paper have some important implications for parasitoid karyology in general. For example, the number of B chromosomes found in *Pnigalio gyamiensis* appeared to be the highest among all other parasitic wasps, although the previous study (Gebiola et al. 2012a) did not reveal B chromosomes in this particular species as well as in *Pnigalio agraules*. This also suggests the importance of karyotypic study of every available population of parasitoid Hymenoptera, even if it apparently belongs to an already examined species (see also Gokhman 2009).
